# Truncal Instability and Titubation in Patients With Acute Encephalopathy With Reduced Subcortical Diffusion

**DOI:** 10.3389/fneur.2021.740655

**Published:** 2021-09-17

**Authors:** Go Kawano, Yukako Yae, Kensuke Sakata, Takaoki Yokochi, Toru Imagi, Keizo Ohbu, Toyojiro Matsuishi

**Affiliations:** ^1^Department of Pediatrics, St Mary's Hospital, Kurume, Japan; ^2^Department of Pediatrics, Kurume University Hospital, Kurume, Japan; ^3^Research Center for Children and Research Center for Rett Syndrome, St Mary's Hospital, Kurume, Japan; ^4^Cognitive and Molecular Research Institute of Brain Diseases, Kurume University, Kurume, Japan

**Keywords:** acute encephalopathy with reduced subcortical diffusion, acute encephalopathy with biphasic seizures and late reduced diffusion, disequilibrium, frontal lobe ataxia, frontopontocerebellar tract, truncal titubation

## Abstract

The present retrospective study aimed to investigate the presence of truncal instability or titubation after the first seizure and second phase in patients with acute encephalopathy with reduced subcortical diffusion (AED). Of the 15 patients with AED who were admitted to our hospital for 3 years and 2 months and had reached developmental milestones for sitting before disease onset, six experienced moderate-to-severe truncal instability while sitting after the first seizure. These patients had a significantly longer first seizure duration and significantly lower GCS scores 12–24 h after the first seizure, as well as significantly higher Tada score and Creatinine and blood glucose levels than those with mild or no truncal instability while in a seated position after the first seizure. Three 1-year-old children with bilateral frontal lobe lesions, particularly in the bilateral prefrontal lobe regions, demonstrated truncal titubation, which has not previously been reported as a clinical feature of AED. Tada score reported to be a predictor of AED prognosis and truncal instability in the sitting position after the first seizure may represent disease severity, but not the specific lesions. Conversely, truncal titubation might be suggestive of bilateral frontal lobe lesions, particularly in patients without severe instability. Further studies on the role of bilateral prefrontal lobe lesions to truncal titubation in patients with AED using more objective evaluation methods, such as stabilometry, are necessary.

## Introduction

Acute encephalopathy with reduced subcortical diffusion (AED) is characterized by the onset of febrile/afebrile seizures and a reduced apparent diffusion coefficient (ADC) in the cortical/subcortical white matter. This is often described as having a “bright tree appearance” on diffusion-weighted (DW) images and appears within 9 days of the first seizure (1–4). Acute infantile encephalopathy predominantly affecting the frontal lobes (AIEF) with a biphasic clinical course and late reduced diffusion in the subcortical white matter was first reported by Yamanouchi et al. and is currently categorized as one of the subtypes of AED (3, 5). AED includes patients with acute encephalopathy with biphasic seizures and late reduced diffusion (AESD), which typically manifests as repetitive and intractable seizures and coma (i.e., the second phase). It is accompanied by the aforementioned “bright tree appearance” 3–5 days after the patient's febrile/afebrile seizure episode (1, 3). As a clinical term, some patients with AED demonstrate a brief seizure as the first episode or do not manifest the second phase. AED mostly affects children of less than school age and has been mainly reported in Japan and East Asia (6). While the mechanism of AED has not yet been clarified, evidence suggests that it may be attributed to late neuronal death triggered by extracellular glutamate stimulation during the first febrile/afebrile seizure (1).

After the first seizure, patients with AED usually show continuous disturbance of consciousness and involuntary stereotypic movements. Instability in a sitting or standing position has also been reported as a neurological symptom (7, 8); however, some patients do not experience apparent disturbance of consciousness or other neurological findings. In patients without obvious neurological findings, a slight degree of instability in a sitting or standing position may suggest the diagnosis of AED.

The primary motor, premotor, and supplementary motor cortices in the frontal lobe are involved in motor action. The prefrontal cortex is also involved in various stages of motor action, such as motor-action initiation, planning, designing, and sequencing, while the frontal lobes control the maintenance of equilibrium (9, 10). Injury to the frontal lobes induces unsteady gait and titubation of the head or trunk, for which damage to the frontopontocerebellar tract is considered to be the most plausible mechanism (11). Although frontal ataxia is common among older adults, this condition is less prevalent among children (10, 12). Further, the cause of instability in a sitting or standing position in patients with AED remains unclear. Hence, the current study aimed to investigate the association between the presence of truncal instability or titubation after the first seizure or second phase and frontal lobe lesions in patients with AED.

## Materials and Methods

### Study Population

**Figure 1:**
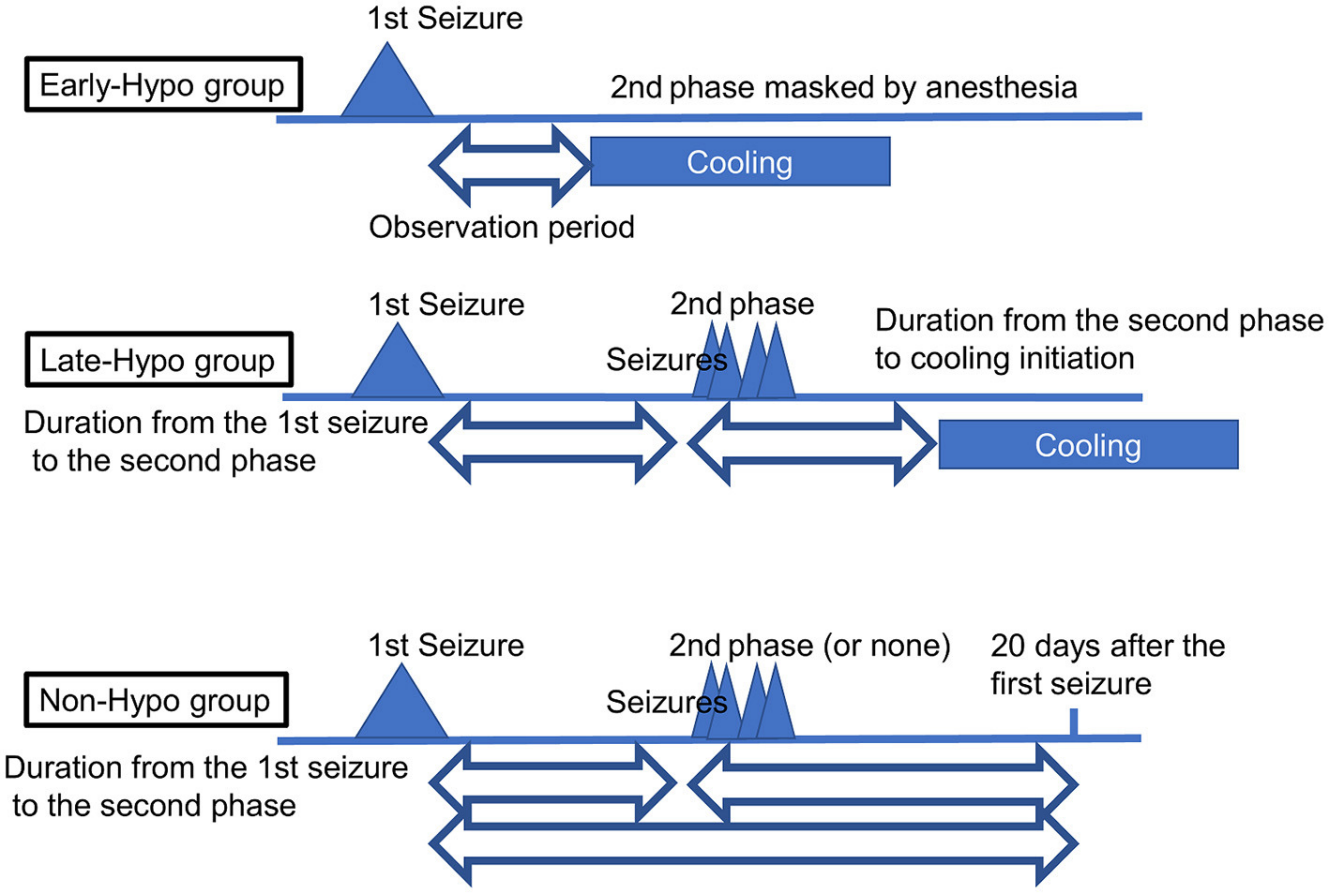
The outlines regarding the observation periods in each treatment group for truncal instability and titubation. In the Early-Hypo group, the observation period for truncal instability and titubation is the period from the 1st seizure to cooling initiation. In the Late-Hypo group, the observation periods are the period from the 1st seizure to the 2nd phase and the period from the 2nd phase to cooling initiation. In the Non-Hypo group, the observation periods are the period from the 1st seizure to the 2nd phase and the period from the 2nd phase to 20 days after the first seizure in patients with the 2nd phase. The observation period is the period from the first seizure to 20 days after the first seizure in patients without the second phase. The observation periods do not include the first 6 h following each seizure.

The medical records of 15 patients with AED (median age = 19.0 months; age range = 11–117 months; five females) who were admitted to our facility between November 2017 and December 2020 were considered in this retrospective study after excluding a 10-month-old patient with insufficient developmental milestones for sitting before the disease onset. We routinely checked for truncal titubation and truncal instability in the sitting or standing position or while walking in all patients who were admitted to our facility after the febrile/afebrile seizure, including those with AED. The second phase was defined as the occurrence of intractable and repetitive seizures and/or deterioration of the consciousness level after a transient improvement in consciousness post febrile status epilepticus. When patients showed truncal instability, excluding the first 6 h following each seizure to exclude the effect of the seizure itself or medications used to terminate the seizure, between the first seizure and initiation of the second phase or 20 days after the first seizure if the second phase did not occur, their condition was classified as follows: severe: inability to sit, stand, or walk even with support; moderate: ability to sit, stand, or walk with support; mild: body swaying despite the ability to continue sitting, standing, or walking without support; or none: no body swaying and the ability to continue sitting, standing, or walking without support. The same classification was applied to patients in the second phase when they showed truncal instability, excluding the first 6 h following each seizure due to the same reasons as mentioned above, between the second phase and the initiation of therapeutic hypothermia or 20 days after the first seizure if therapeutic hypothermia was not performed. Depending on whether patients underwent therapeutic hypothermia, they were classified into the following groups: patients who started undergoing therapeutic hypothermia administered by an attending physician before the second phase because of a worsening level of consciousness or continuously impaired consciousness (Early-Hypo group); patients who started undergoing therapeutic hypothermia after the initiation of the second phase (Late-Hypo group); and patients who did not undergo therapeutic hypothermia (Non-Hypo group). The outlines regarding the observation periods in each treatment group for truncal instability and titubation are shown in Figure 1. Patients underwent therapeutic hypothermia; however, some patients did not undergo therapeutic hypothermia because of either the mildness of clinical neurological presentation or absence of the second phase. The methods have been described in our previous report (4).

Ten of the 15 patients in the present study were included in our previous investigation that aimed to elucidate the determinants of outcomes for AED (4). The current study was approved by the institutional review board (IRB) of St Mary's Hospital, Fukuoka, Japan (IRB number: 20-0601). All methods used were consistent with relevant guidelines and regulations. Informed consent was obtained from all participants and/or their legal guardians. Moreover, written informed consent was obtained from the legal guardians of two patients with truncal titubation for the use of their videographic images.

### Data Collection

The following clinical data were collected from the medical records of each patient: age; sex; associated infections; first seizure duration; Glasgow Coma Scale (GCS) scores 12–24 h after the first seizure; treatment group (Early-Hypo, Late-Hypo, or Non-Hypo); presence of truncal titubation and instability in a sitting or standing position or while walking, excluding the first 6 h following each seizure, between the first seizure and the initiation of the second phase [all patients in the Late-Hypo group and three patients (No. 1, 2, and 7) in the Non-Hypo group], between the first seizure and the initiation of therapeutic hypothermia [all patients in the Early-Hypo group (No. 8 and 14)], or 20 days after the first seizure in the absence of the second phase [three patients (No. 9, 11 and 13) in the Non-Hypo group]; presence of truncal titubation or instability in a sitting or standing position or while walking, excluding the first 6 h following each seizure, between the initiation of the second phase and therapeutic hypothermia (all patients in the Late-Hypo group) or 20 days after the first seizure if therapeutic hypothermia was not applied [three patients [(No. 1, 2, and 7) in the Non-Hypo group]; days when reduced subcortical ADC was confirmed on magnetic resonance (MR) images after the first seizure; distribution of brain lesions on MR images [bilateral/unilateral frontal, parietal, temporal or occipital lobe(s)]; presence of diffuse lesions with injury around the perirolandic regions on MR images of at least 1 hemisphere; presence of lesions on basal ganglia/thalamus as shown by MR images; serum levels of aspartate aminotransferase (AST), alanine aminotransferase (ALT), lactate dehydrogenase, blood urea nitrogen (BUN), creatinine (Crea), and glucose after the first seizure; cell count and protein level in the cerebrospinal fluid (CSF); Tada scores after the first seizure (13); use of additional treatments, such as antiepileptic medications for seizure termination or prevention, intravenous methylprednisolone pulse therapy, or immunoglobulins (1 g/kg/dose); and clinical outcomes. GCS score has been included to examine the relationship between the consciousness level after the first seizure and truncal instability or titubation. In addition, the consciousness level was included in the Tada score (13), which is a predictor of AESD, and loss of consciousness 24 h after onset and prolonged seizure at onset were reported to be poor predictors of AED (3). Tada score is a predictor of AESD for patients with febrile seizures and consists of seven variables: consciousness level, age, duration of convulsions, enforcement of mechanical intubation, AST level, glucose level, and Crea level (13). Four clinical psychologists who were unaware of the treatment assignment assessed the neurodevelopmental outcomes, and two pediatricians independently assessed the neurological outcomes of each patient at 12 months after disease onset, excluding one patient (No. 9) whose outcome was assessed at 6 months after the onset because of an insufficient observational period, on the basis of medical records and scored them using the Pediatric Performance Category Scale (PCPC). PCPC scores of 1–6 represent normal, mild, moderate disabilities, severe disabilities, coma or vegetative state, and death, respectively (14). In the event of a conflict regarding PCPC scores, the two pediatricians reached a consensus after discussion (4).

### Statistical Analysis

SPSS (version 25; IBM, Armonk, NY, USA) was used to conduct all statistical analyses. We compared the clinical features between patients with and without moderate-to-severe truncal instability in a seated position after the first seizure using the Mann-Whitney U test (two-tailed) for numerous variables or Fisher's exact test (two-tailed) for categorical and discrete variables with effect sizes of *r* and *phi* for 2 × 2 tables or the Cramer's *V* for those other than 2 × 2 tables. Statistical significance was set to *P* < 0.05.

## Results

### Clinical Characteristics

The clinical characteristics of the sample are shown in Tables 1, 2. All patients except one (patient No. 6) were prescribed fosphenytoin (fPHT) after the first seizure or in the second phase for seizure prevention without affecting consciousness during the acute phase. The serum phenytoin concentration was not measured in each patient. We did not routinely check for the presence of anti-N-methyl-D-aspartate receptor or anti-myelin oligodendrocyte glycoprotein antibodies in the CSF of each patient because none with AED had neuroimaging findings suggestive of acute disseminated encephalomyelitis or multiple sclerosis as defined by the criteria of the International Pediatric Multiple Sclerosis Group in 2013, such as multiple high-intensity lesions in ADC, or clinical features typical of autoimmune encephalitis, such as orofacial or limb dyskinesias, choreoathetosis, or dysautonomia. The occurrence of an associated infection was identified in 10 patients. Preceding viral infections were confirmed on the basis of positivity on at least one antigen test or significantly increased antigen titers in paired serum samples. Although we confirmed the absence of herpes simplex virus (HSV) infection in each patient by examining their CSF samples for HSV deoxyribonucleic acid or serum samples for anti-HSV immunoglobulin G, we did not routinely conduct CSF viral culture or polymerase chain reaction test for the detection of other viruses in each patient; the CSF samples of 10 of the 15 patients with AED (patient No. 1, 2, 4, 7–11, 13, and 14) were examined. The results were normal in all patients except two; patient No. 2 had a slightly elevated cell count (9/μL), and patient No. 9 had a highly elevated cell count with septic meningitis (3,515/μL).

**Table 1:**
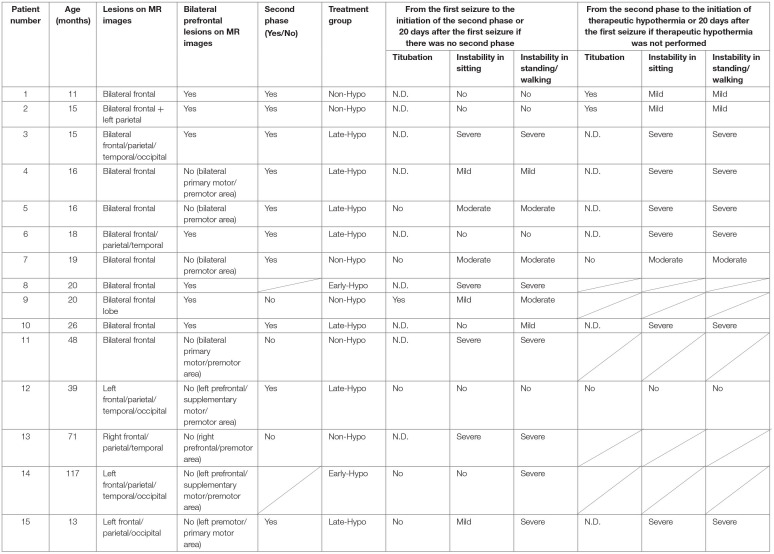
Clinical characteristics of the sample.

*MR, magnetic resonance; N.D., not determined*.

**Table 2:**
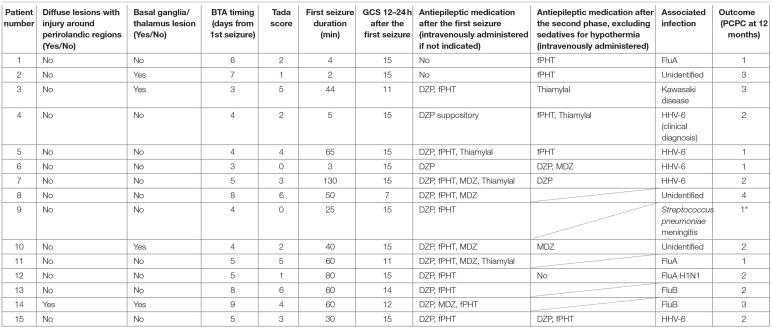
Additional clinical characteristics of the sample.

*PCPC at 6 months after disease onset. BTA, bright tree appearance on diffusion weighted image; DZP, diazepam; fPHT, fosphenytoin; GCS, Glasgow Coma Scale, FluA/B, Influenza virus A/B; HHV-6, human herpesvirus 6; MDZ, midazolam.

**Figure 2:**
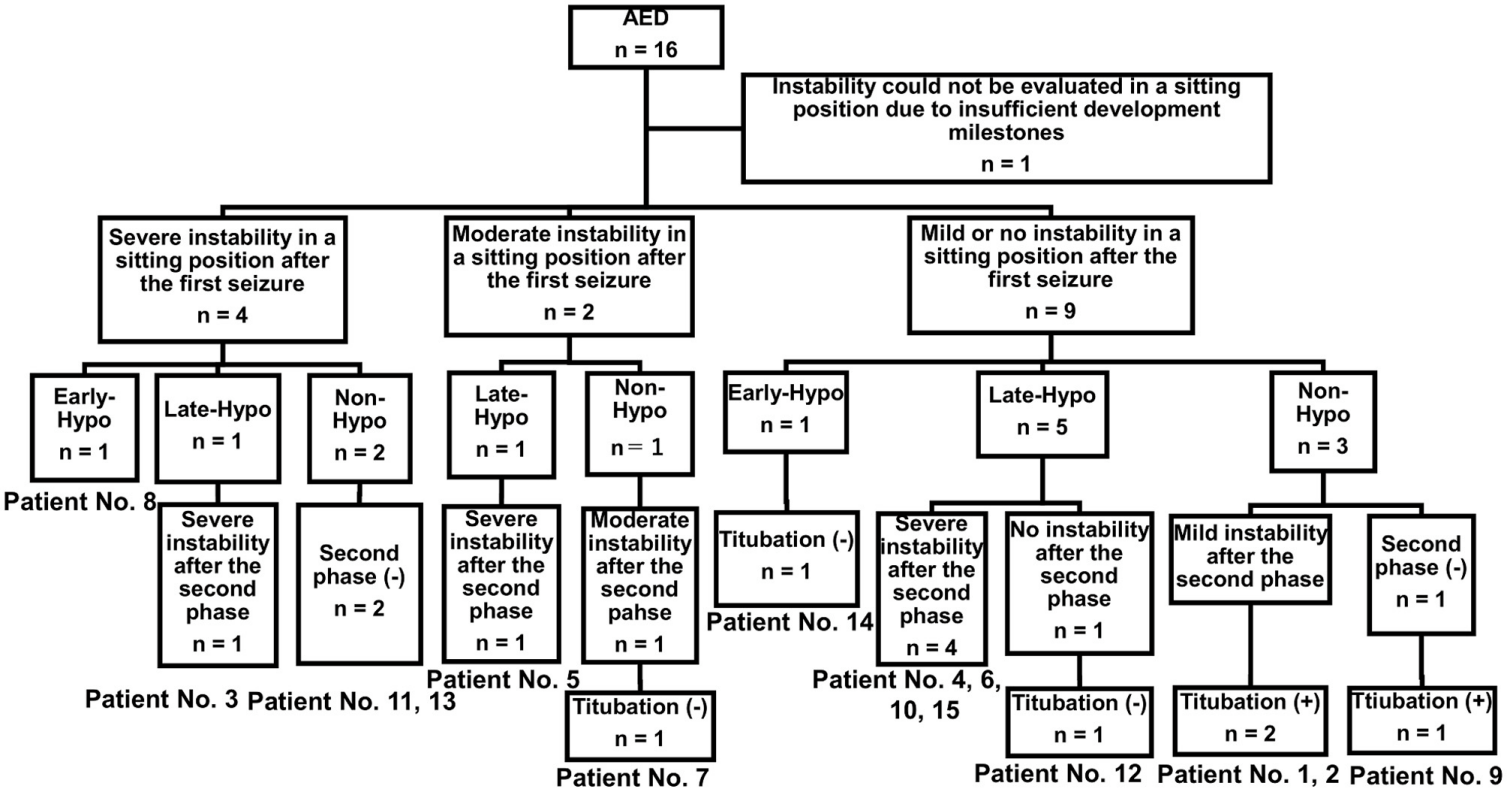
Study sample. Four of the 15 patients with AED admitted to our hospital during the study period had presented severe truncal instability in the sitting position after the first seizure (patient No. 3, 8, 11, and 13). Five of the 15 patients with AED admitted to our hospital during the study period had presented severe truncal instability in the sitting position after the second phase (patient No. 4, 5, 6, 10, and 15). Titubation could not be determined in these patients because of severe instability in sitting. One of the two patients with AED who showed moderate instability in a sitting position after the first seizure did not undergo therapeutic hypothermia and experienced no truncal titubation in the sitting position after the second phase (patient No. 7). Three of the nine patients with AED who had mild or no truncal instability in the sitting position after the first seizure did not undergo therapeutic hypothermia and had truncal titubation in the sitting position after the first seizure (patient No. 9) or the second phase (patient No. 1 and 2). AED, acute encephalopathy with reduced subcortical diffusion.

Nine of the 15 patients underwent therapeutic hypothermia (Table 1, Figure 2). Patients were classified into the Early-Hypo group (*n* = 2), Late-Hypo group (*n* = 7), or Non-Hypo group (*n* = 6). Therapeutic hypothermia was not performed in patients with mild clinical neurological presentation (*n* = 3, patient No. 1, 2, and 7) or absence of the second phase (*n* = 3, patient No. 9, 11, and 13). None of the patients in the Non-Hypo group were intubated, mechanically ventilated, infused continuously with sedatives, or subjected to body-temperature maintenance using a cooling device. Except for the administration of rectal diazepam suppository (0.5 mg/kg/dose) or bolus injection of diazepam (0.3–0.5 mg/kg/dose), midazolam (0.1–0.4 mg/kg/dose), or thiamylal (3–5 mg/kg/dose) to terminate seizures or sedate patients before neuroimaging acquisition, mechanical ventilation, or therapeutic hypothermia, none of the patients received medications, such as intravenous phenobarbital or continuous infusion of midazolam, that affected their consciousness (Table 2). The first dose of fPHT was administered at a concentration of 22.5 mg/kg infused for over 7.5 min; the concentrations of subsequent doses administered for 7.5 min every 24 h thereafter were decreased to 7.5 mg/kg. None of the patients received steroid pulse therapy or immunoglobulins, except patient No. 3 who received immunoglobulins (1 g/kg/dose) for the treatment of Kawasaki disease.

### Frontal Lesions and Truncal Instability and Titubation in Sitting After the First Seizure and Second Phase

**Figure 3:**
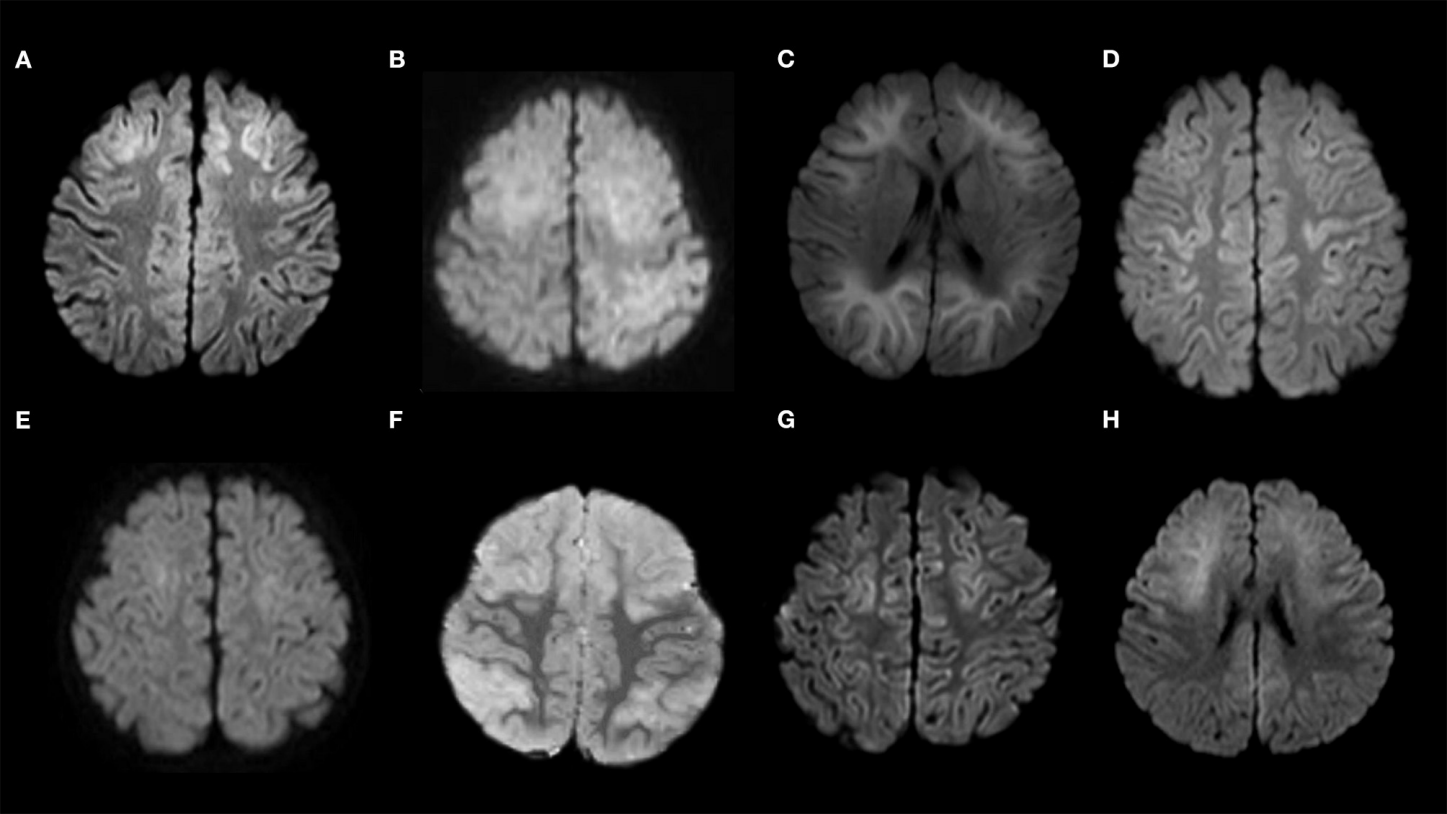
Diffusion-weighted images of the study sample. **(A)** Patient No. 1; **(B)** Patient No. 2; **(C)** Patient No. 3; **(D)** Patient No. 4; **(E)** Patient No. 5; **(F)** Patient No. 6; **(G)** Patient No. 7; **(H)** Patient No. 8. Patients 1–8 show bilateral frontal lesions. Patients 4, 5, and 7 do not show bilateral prefrontal lesions.

**Figure 4:**
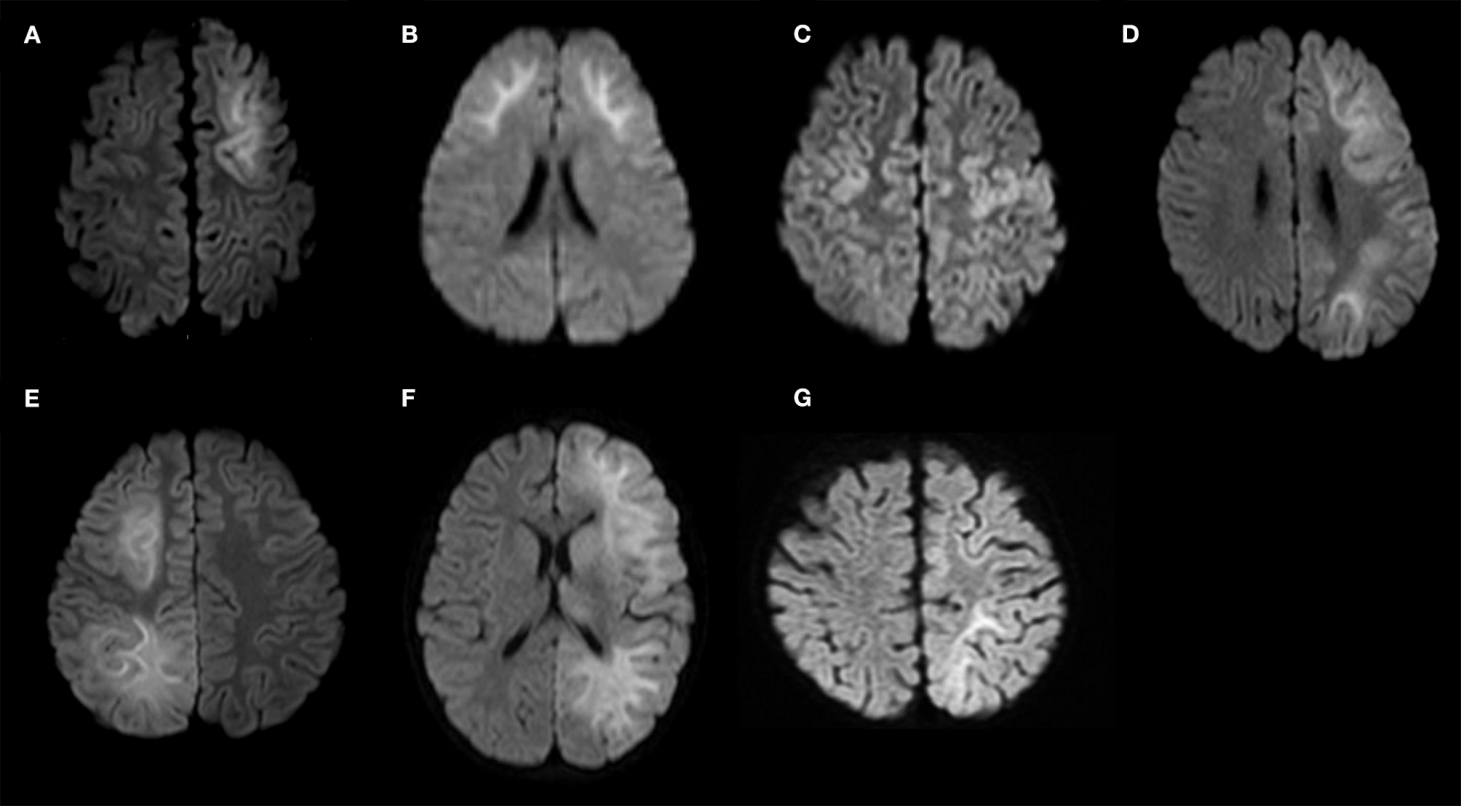
Diffusion-weighted images of the study sample (continued). **(A)** Patient No. 9; **(B)** Patient No. 10; **(C)** Patient No. 11; **(D)** Patient No. 12; **(E)** Patient No. 13; **(F)** Patient No. 14; **(G)** Patient No. 15. Patients 9–11 show bilateral frontal lesions. Patient No. 11 does not show bilateral prefrontal lesions. Patients 12–15 show unilateral frontal lesions.

DW images of the 15 patients, which were acquired 3–9 days after the first seizure, are shown in Figures 3A–H, 4A–G.

Regarding truncal instability and titubation after the first seizure in the 11 patients with bilateral frontal lesions, eight (five with bilateral prefrontal lesions and three without bilateral prefrontal lesions) did not present severe instability while sitting (patient No. 1, 2, 4–7, 9, and 10, Table 1, Supplementary Table 1, Supplementary Figure 1). While one of these eight patients was confirmed to have bilateral prefrontal lesion and truncal titubation (patient No. 9), and two were confirmed to lack any bilateral prefrontal lesion or any signs of truncal titubation (patient No. 5 and 7), whether the other five developed truncal titubation was not confirmed (patient No. 1, 2, 4, 6, and 10) because they presented to our hospital after the second phase. Of the four patients without bilateral frontal lesions, three had unilateral prefrontal lesions (patient No. 12, 13, and 14), and one did not have any unilateral prefrontal lesions (patient No. 15). Of the four patients without bilateral frontal lesions, three lacked severe instability in the sitting position and truncal titubation (patient No. 12, 14, and 15).

Regarding truncal instability and titubation after the second phase in the 8 patients with bilateral frontal lesions and the second phase, excluding five patients in whom truncal titubation could not be determined because of severe instability in sitting after the second phase (patient No. 3, 4–6, and 10, Supplementary Figure 2), two with bilateral prefrontal lesions were confirmed to have truncal titubation (patient No. 1 and 2), and one without bilateral prefrontal lesions was confirmed not to have truncal titubation (patient No. 7). Of the two patients with the second phase and without bilateral frontal lesions, one with a unilateral prefrontal lesion did not have truncal titubation (patient No. 12); truncal titubation was not determined for the other patient without a unilateral prefrontal lesion because of the severe instability in sitting (patient No. 15).

### Comparison of Clinical Features Between Patients Without and With Moderate-to-Severe Truncal Instability in the Sitting Position After the First Seizure

Patients with moderate-to-severe truncal instability in the sitting position after the first seizure had significantly longer first seizure duration and lower GCS scores 12–24 h after the first seizure, as well as higher Tada score and Crea and blood glucose levels than those with mild or no truncal instability in the sitting position after the first seizure (*P* = 0.025, 0.020, 0.002, 0.022, and 0.020, respectively, Table 3). There were no significant differences in the other measured clinical features between the two groups (Table 3).

**Table 3:** Comparison of the clinical features of patients without and with moderate-to-severe instability in the sitting position after the first seizure.

	**Mild or no instability in the sitting position after the first seizure**			**Moderate-to-severe instability in the sitting position after the first seizure**			**Effect size**	***p* value**
	***n*** **=** **9**			***n*** **=** **6**				
Age in months, median (IQR), n	18	(14–32.5)	9	19.5	(16–48)	6	0.168	0.546
Sex, female (%)	4	(44.4)		1	(16.7)		0.289	0.580
GCS, median (IQR), *n*	15	(15–15)	9	12.5	(11–15)	6	0.580	0.020^*^
Duration of seizure in min, median (IQR), *n*	27.5	(4.5–50)	9	60	(50–65.5)	6	0.580	0.025^*^
Truncal titubation *n* (%)	3	(33.3)		0	(0)		0.408	0.229
Distribution of the lesion								
Bilateral frontal, *n* (%)	6	(66.7)		5	(83.3)		0.185	0.604
Bilateral prefrontal, *n* (%)	5	(55.6)		2	(33.3)		0.218	0.608
Diffuse lesion including perirolandic area, *n* (%)	1	(11.1)		0	(0)		0.218	1.000
Basal ganglia or thalamus lesion, *n* (%)	3	(33.3)		1	(16.7)		0.185	0.604
Tada score, median (IQR), *n*	2	(1–2.5)	9	5	(4–6)	6	0.768	0.002^*^
Patients with biphasic clinical course, *n* (%)	7	(77.8)	9	3	(75.0)	4	0.318	0.510
AST, IU/L, median (IQR), *n*	38.0	(32.0–55.0)	9	49.5	(39.0–67.0)	6	0.320	0.237
ALT, IU/L, median (IQR), *n*	13.0	(10.5–17.5)	9	17.0	(16.0–21.0)	6	0.366	0.168
LDH, IU/L, median (IQR), *n*	333.0	(294.5–338.5)	9	336.0	(293.0–390.0)	6	0.198	0.475
BUN, mg/dL, median (IQR), *n*	8.4	(6.85–12.8)	9	11.95	(10.7–14.4)	6	0.365	0.171
Creatinine, mg/dL, median (IQR), *n*	0.25	(0.21–0.31)	9	0.34	(0.31–0.47)	6	0.581	0.022^*^
Blood glucose, mg/dL, median (IQR), *n*	156.5	(130.0–200.5)	9	258.0	(237.0–271.0)	6	0.600	0.020^*^
fPHT administration after the first seizure, *n* (%)	5	(50.0)		5	(77.8)		0.289	0.580
Therapeutic options							0.218	0.790
Early-Hypo group, *n* (%)	1	(16.6)		1	(11.1)			
Late-Hypo group, *n* (%)	5	(50.0)		2	(44.4)			
Non-Hypo group, *n* (%)	3	(33.3)		3	(44.4)			
PCPC 12 months after disease onset	2	(1.5–2.5)	9	2.5	(1–3)	6	0.191	0.514

* *P < 0.05. AST, aspartate aminotransferase; ALT, alanine aminotransferase; BUN, blood urea nitrogen; fPHT, fosphenytoin; GCS, Glasgow Coma Scale; IQR, interquartile range; LDH, lactate dehydrogenase; PCPC, Pediatric Performance Category Scale*.

### Truncal Titubation in Three Patients With Bilateral Prefrontal Lesions

Three of the nine patients with AED who had mild or no truncal instability in the sitting position after the first seizure had truncal titubation in the sitting position (patient No. 1, 2, and 9, Figure 2). Patient No. 1 (Table 1) was a previously healthy 11-month-old boy and had his first seizure lasting for 4 min on the second day of fever. He was discharged the same day after being diagnosed with a febrile seizure with normal neurological findings and blood test results. He had the second seizure 4 days after the first seizure. He had two similar seizures within 6 h of the second seizure, which lasted for 30 s, and his level of consciousness was clear during the interictal periods. The patient did not present with muscle weakness or cerebellar symptoms, such as dysmetria, intention tremor, or nystagmus. After the fourth seizure, the patient was intravenously administered fPHT at a dose of 22.5 mg/kg and admitted to our hospital. fPHT administration was continued with 7.5 mg/kg/dose every 24 h thereafter until the eighth day after the first seizure, when fPHT was switched to oral levetiracetam. He was confirmed to have truncal titubation on admission to the pediatric ward, which was 3 h after the first fPHT administration. He showed horizontal, mainly backwards-and-forwards and occasionally left-and-right, truncal titubation (low-frequency, 1 Hz; small amplitude, 5°) and trivial head titubation (low-frequency, 2 Hz; small amplitude) in the sitting position (the frequency and amplitude were measured using recorded video images and a protractor on the screen, Supplementary Video 1). This was continuously observed until 12 days after his first seizure. The results of blood and CSF tests at admission were all within the normal range. DW images acquired on the sixth day after the first seizure revealed a bright tree appearance in the bilateral frontal lobes, especially in the bilateral prefrontal lobe regions (Figure 3A). Subsequent neurological examination revealed no remarkable changes in the patient's neurodevelopmental milestones before and after the disease onset; the patient walked with support, shook hands, and spoke a few meaningful words. Sleep electroencephalography (EEG) on the eighth day after the first seizure showed no remarkable findings, and an MR image (DW image) acquired on the seventeenth day revealed slight atrophy in the bilateral hemispheres without high-intensity lesions.

Patient No. 2 (Table 1) was a previously healthy 14-month-old girl who experienced a generalized clonic seizure lasting for 2 min on the second day of fever and was brought to a referring hospital. She had right-sided hemiplegia, which lasted for 1 h. Activity in her right hand was reduced, but she was able to maintain a sitting position without support. Her white blood cell count (19.0 × 10^9^/L with 82.9% polymorphonuclear leukocytes) and C reactive protein level (2.7 mg/dL) were within the normal range. On the third day after the first seizure, her fever abated, and she was diagnosed with exanthema subitum. On the fifth day after the first seizure, she had recurrent tonic-clonic seizures of the right hand with a deviation of the eyes. Intravenous fPHT was administered at a dose of 22.5 mg/kg to terminate the seizure. The EEG performed on the next day showed frequent sharp waves in the left hemisphere. MR images (DW image) acquired on the eighth day after the first seizure revealed a bright tree appearance in the bilateral frontal lobes, especially in the bilateral prefrontal lobe regions, bilateral globus pallidus, and caudate nucleus (Figure 3B, Supplementary Figure 3). The patient was transferred to our facility on the same day for further management of the disease. Her level of consciousness was normal with a GCS score of 15 on admission and showed truncal titubation with small-amplitude oscillation—mainly left-and-right truncal movements (low-frequency, 1 Hz; relatively large amplitude, 15°)—in the sitting position (the frequency and amplitude were measured using recorded video images and a protractor on the screen, Supplementary Video 2). This was continuously observed from the day of admission to our hospital until 19 days after her first seizure. Although the patient's parents reported that it had not been observed before the first seizure, we could not confirm the time at which truncal titubation began because the patient was transferred to our hospital after the second seizure. The activity of the right hand was reduced; the patient could not perform a pincer grasp with the right hand and preferred to use her left hand. She did not show forced hand grasping. Except for truncal titubation, neurological findings were normal. We observed no muscle weakness or cerebellar symptoms, such as dysmetria, intention tremor, or nystagmus on admission. CSF examination revealed an increased number of cells (9/μL; lymphocytes, 100%) and normal protein levels (20 mg/dL). EEG performed on the ninth day showed sharp waves in the bilateral frontal and right temporal regions. The patient was intravenously administered fPHT at a dose of 22.5 mg/kg on that day and at 7.5 mg/kg/dose every 24 h thereafter until 12 days after the first seizure when fPHT was switched to oral levetiracetam. The bright tree appearance disappeared from the DW images on the seventeenth day after the first seizure, but the bilateral globus pallidus lesions remained and atrophy in both hemispheres was observed on MR images. She was able to pull herself up and grab toys using her right hand, but her preference for using her left hand remained at her discharge from the hospital 26 days following the first seizure.

Patient No. 9 (Table 1) was a previously healthy 20-month-old boy and had the first seizure on the second day of fever. He was brought to our hospital, and the seizure lasted for 25 min until intravenous diazepam was administered. The blood test on arrival revealed increased white blood cell count (14.6 × 10^9^/L with 85.8% polymorphonuclear leukocytes) and C reactive protein level (11.5 mg/dL). CSF examination revealed an increased number of cells (3,515/μL; lymphocytes, 2%) and protein levels (401 mg/dL). The patient was diagnosed with bacterial meningitis, and dexamethasone 0.6 mg/kg/day for 2 days and antibiotics (meropenem and ceftriaxone) were started. The CSF culture revealed *S. pneumoniae*, and antibiotics were switched to ampicillin following the susceptibility test results until the fourteenth day after the first seizure. The patient was also administered fPHT intravenously at a dose of 22.5 mg/kg after the first seizure and 7.5 mg/kg/dose every 24 h thereafter until the fourth day after the first seizure when fPHT was switched to oral levetiracetam. He started waking up at 16 h after the first seizure and kept sitting on the bed with continuous truncal titubation, which was mainly backwards-and-forwards (low-frequency, 1 Hz; small amplitude). He had truncal titubation for 3 days. DW images acquired on the fifth day after the first seizure revealed a bright tree appearance in the bilateral frontal lobes, especially in the bilateral prefrontal lobe regions (Figure 4A). Subsequent neurological examination revealed no remarkable changes in the patient's neurodevelopmental milestones before and after disease onset except his gross motor development milestone; the patient was only able to walk with support at his discharge from the hospital 26 days following the first seizure.

## Discussion

Upon evaluating the presence of truncal instability and titubation in patients with AED, the present study revealed two major findings. First, six of the 15 patients with AED had moderate-to-severe truncal instability in a sitting position after their first seizures, and these patients had significantly longer first seizure duration and significantly lower GCS scores 12–24 h after the first seizure, as well as significantly higher Tada score and Crea and blood glucose levels than those with mild or no truncal instability in the sitting position after the first seizure. Second, bilateral prefrontal lobe regions were observed in three 1-year-old children with truncal titubation (patient No. 1, 2, and 9).

### Truncal Instability and Titubation After the First Seizure or the Second Phase in Patients With AED

Most of the patients (14 out of 15 patients) with AED had mild-to-severe truncal instability in the sitting position or while standing/walking after the first seizure or the second phase. While the inability to sit or stand was reported to be a symptom of AED before the second phase in four out of 32 patients (12.5%) with AED (8), nine out of 15 patients (60%) in our current study had mild-to-severe truncal instability while in a sitting position after the first seizure. Our higher prevalence for truncal instability may be attributed to the fact that we routinely checked for truncal titubation and truncal instability in a sitting or standing position or while walking in all patients who were admitted to our facility after the febrile/afebrile seizure, including all patients with AED. In addition to major neurological findings reported in patients with AED before the second phase, such as somnolence, slight disturbance of consciousness, involuntary movements (e.g., dystonia, oral dyskinesia, stereotyped movement), slight hemiplegia, sensory disturbance or hemianopsia (7, 8), instability may be an important clinical sign for suspecting AED after the first seizure and the second phase.

The duration and timing for which patients with AED showed truncal instability or titubation has not been reported previously. Thus, we set the evaluation period for truncal instability and titubation to 20 days after the first seizure in the Non-Hypo group because titubation was identified after the first seizure or the second phase and was observed until 12 days after the first seizure in patient No. 1, 19 days in patient No. 2, and 3 days in patient No. 9 in the current study. Conversely, we excluded the period after therapeutic hypothermia in the Early-Hypo and Late-Hypo groups for observing truncal instability and titubation to exclude the effects of prolonged sedation use for therapeutic hypothermia. It is also important to note that it should be evaluated several hours after a seizure or antiepileptic medication use (e.g., diazepam, midazolam) to exclude the effects of antiepileptic medications.

The Tada score was significantly higher among patients with moderate-to-severe truncal instability in a sitting position after the first seizure than those with mild or no instability. The comparison of clinical features between patients with and without moderate-to-severe truncal instability in a sitting position was performed because the median age of our patients was 19 months; hence, judging instability while the patient stood/walked was considered difficult in some cases due to individual developmental milestones not having been reached before the onset of the disease. As shown by multivariate analysis in a previous study, the Tada score and basal ganglia/thalamic lesions were the determinants of poor outcomes in patients with AED (4). Therefore, the presence of moderate-to-severe truncal instability may also be a sign that is associated with the Tada score and may suggest poor outcomes in patients with AED, although there was no significant difference in the direct comparison of outcomes between patients with and without moderate-to-severe truncal instability in a sitting position in the current study, presumably, because of the difference in therapeutic options.

### Truncal Titubation and Bilateral Prefrontal Lesions

Of the 15 patients with AED who were included in our current study, three 1-year-old children with AED had truncal titubation and bilateral prefrontal lesions. The patient who did not have severe truncal instability in a sitting position after the second phase did not show truncal titubation (patient No. 12), but did have unilateral hemispheric lesions, including unilateral prefrontal lesions. This observation was consistent with a previous report on a patient with truncal titubation and lesions of the bilateral frontal lobes, especially in Brodmann area 10 and the origin of the frontopontocerebellar tract, causing frontal lobe ataxia (10). This may suggest that bilateral prefrontal lesions are responsible for truncal titubation in patients with AED, although this hypothesis was based on findings collected from a small sample. According to a previous report, although patients with AIEF do not have motor paralysis and are able to behave in response to brief commands from others, stereotypic movements (e.g., tapping on one hand, sucking the hands and then throwing the arms forward repeatedly, jumping on the bed, and catalepsy) were reported after the recovery of consciousness along with the regression of verbal function and marked diminution of active and voluntary behavior (5); however, truncal titubation has not been reported previously.

We administered fPHT to all three patients with truncal titubation without measuring their serum phenytoin concentrations; however, phenytoin toxicity was not likely to be the cause of truncal titubation. Patient No. 1 was confirmed to have truncal titubation 3 h after the first fPHT administration; patient No. 2 had already shown truncal titubation upon her admission, 3 days following her first and also last receipt of fPHT after the first seizure; and patient No. 9 was confirmed to have truncal titubation 20 h after the first fPHT administration. fPHT is completely converted to phenytoin following intravenous administration, with a half-life of a median of 8 min (4–16 min) and mean total phenytoin half-life values following fPHT administration of 12.0–28.9 h (15–18). None of the three patients had a renal or hepatic disease or hypoalbuminemia, which decrease the ability to bind fPHT and elevate the plasma concentrations of unbound phenytoin (17, 19). There was no concomitant use of other medications that decreased phenytoin metabolism, such as valproic acid or cimetidine, in these patients. Further, the earliest signs of phenytoin toxicity are horizontal nystagmus, ataxia, and drowsiness, although there is large individual variability in the correlation between serum concentrations and clinical findings (20, 21). None of our patients with truncal titubation had nystagmus or drowsiness.

Cerebellar, vestibular, or frontal lobe disorders are usually responsible for ataxia. Head titubation is reported as one of the cerebellar manifestations. Horizontal, high-frequency (3 Hz), small-amplitude (5–10°) titubation with a variable duration ranging from several min to 1 h in 13 children with Joubert syndrome has previously been reported, although the pathogenesis of head titubation is unclear (22). Patients with rhombencephalosynapsis also demonstrate intermittent or almost constant head-shaking movements with a lower frequency and larger amplitudes than those with Joubert syndrome (23). Head-shaking in rhombencephalosynapsis represents a response to a deficit in central vestibular processing or an underlying rhythmic motor pattern that is usually suppressed in the presence of appropriate vestibular feedback (23). Furthermore, head titubation has been reportedly observed in patients with Dandy–Walker syndrome, which involves the partial midline fusion of the cerebellar hemispheres with vertical folia (24).

Titubation is also one of the clinical signs of frontal lobe ataxia. Patients with frontal lobe ataxia have been reported since the late 19^th^ century, and MRI and other neuroimaging modalities have revealed the pathology of the frontal lobe ataxia (10, 11, 25, 26). The frontal lobes control truncal motion, postural responses, maintenance of equilibrium, and locomotion (10). Disequilibrium attributable to frontal lobe lesions manifests as a wide-stance base, increased body sway, falls after minor perturbations, poor truncal control, locomotor disability with gait ignition failure, shuffling, and freezing without showing signs of cerebellar ataxia (e.g., dysmetria, asynergy of limb movement, dysarthria, and nystagmus) (10). Initially, frontal ataxia was reported in patients with injury to the bilateral frontal lobes, particularly the prefrontal cortex (Brodmann area 10) or the supplementary motor area (11, 26). Later, three pediatric patients with unilateral frontal lobe injury, particularly in the right middle or upper frontal gyri, were reported to show disequilibrium and/or mild signs of upper limb ataxia (12). Injury to the frontopontocerebellar tract is the most plausible mechanism for frontal lobe ataxia (11).

We were unable to ascertain the exact time points at which patients No. 1 and 2 first presented truncal titubation because both were admitted to our facility after the initiation of the second phase. The frequency and amplitude seen in our three patients with truncal titubation differed from those reported in patients with Joubert syndrome (22). The frequency was 1–2 Hz, which was slower than that reported in patients with Joubert syndrome. These three patients had a GCS score of 15 and a Tada score <3, and they were able to sit alone and stand with support, which suggests that they had a milder clinical course than the other patients in our study (Tables 1, 2).

Truncal instability in a sitting position or while walking after the first seizure was also seen in patients with AED without bilateral prefrontal lesions (patient No. 4, 5, 7, 11, and 13–15). Patients with AED do not typically show any abnormal neuroimaging findings in the cerebellum or brain stem. Furthermore, parietal lobe injury was reported to cause ataxia (27, 28). Hence, truncal instability in a sitting or standing position or while walking may be attributed to frontal, parietal, basal ganglia or thalamic lesions because none of the patients in our current study had any abnormal findings in the cerebellum or brain stem. Unilateral frontal lobe injury and disequilibrium were previously reported in three pediatric patients (12). Three out of four patients with AED without bilateral frontal lesions in our present study showed instability (patient No. 13-15, Table 1), but none of the patients with AED without bilateral frontal lesions showed truncal titubation. Our data suggest that truncal titubation may not stem only from disequilibrium but may also be related to other mechanisms in bilateral prefrontal lesions. Because our study included a small study sample, further studies investigating the association of the bilateral prefrontal lobe lesions with truncal titubation in patients with AED must be conducted using more objective methods, such as stabilometry or quantitative evaluation with oculomotor and vestibular testing (29, 30).

### Limitations

First, it is difficult to conclude the relationship between truncal titubation and prefrontal lesions because of the following reasons. This study included a small number of cases with titubation. Although MR images were acquired 3–9 days after the first seizure in our study and all patients without bilateral prefrontal lesions (patient No. 4, 5, 7, 11, 12–15) undertook MR imaging 4 days after onset, some patients might have had lesions broader than their MR images at peak stages of the disease because it has been reported that lesions in the bilateral frontal and occipital regions seen on the first MRI on the day after onset spread to the entire subcortical white matter on the second MRI taken 5 days after onset (31). The EEG findings of increased regions with sharp waves in patient No. 2 in our study may also suggest that the damaged lesions spread to the bilateral frontal and right temporal regions after the first EEG. In addition, we could not exclude the possibility that medications used to terminate seizure could influence truncal instability and titubation although none of the patients received medications, such as intravenous phenobarbital or continuous infusion of midazolam, that affected their consciousness except for the administration of diazepam, midazolam, or thiamylal to terminate seizures or sedate patients prior to neuroimaging acquisition, mechanical ventilation, or therapeutic hypothermia.

It is important to investigate the relationship between truncal titubation and prefrontal lesions in future studies because truncal titubation would be easier to identify for clinicians when the patient continues to sit or stand compared to neurological findings reported in patients with AED before the second phase, such as somnolence, slight disturbance of consciousness, involuntary movements (e.g., dystonia, oral dyskinesia, stereotyped movement), slight hemiplegia, sensory disturbance, or hemianopsia (7, 8) particularly, when a patient is under 3 years of age [i.e., the most common age at which children develop AED (6)]. In addition, further studies investigating truncal instability and titubation as early clinical markers of AED before and after the second phase are necessary because a proposed AESD predictive score showed a low positive predictive value of 47% (32).

Second, this study was performed retrospectively, and truncal titubation that occurred for only a few hours may have been missed; however, as the presence of truncal titubation and instability in a sitting position or while walking in patients who were admitted to our hospital with seizures were routinely checked and recorded every day, we minimized the risk of overlooking an instance of titubation or instability.

Third, although physical examination related to cerebellar symptoms, such as dysmetria, intention tremor, and nystagmus, were routinely assessed, the quantitative evaluation of oculomotor and vestibular systems was not performed to differentiate the cerebellar diseases or vestibular lesions in each patient, as 11 of the total 15 patients were under the age of 3 years and were uncooperative, particularly after the first seizure.

### Conclusion

Despite the limited ability to extrapolate findings from a small number of cases, truncal instability in a sitting position after the first seizure in patients with AED may represent disease severity, not specific lesions in the brain. The fact that three patients with AED had continuous truncal titubation in the sitting position implies that truncal titubation in AED may be related to the presence of bilateral frontal lobe lesions; further prospective studies are needed to confirm this.

## Data Availability Statement

The raw data supporting the conclusions of this article will be made available by the authors, without undue reservation.

## Ethics Statement

The studies involving human participants were reviewed and approved by The institutional review board of St Mary's Hospital, Fukuoka, Japan (IRB number: 20-0601). Written informed consent from the participants' legal guardian/next of kin was not required to participate in this study in accordance with the national legislation and the institutional requirements. Written informed consent was obtained from the minor(s)' legal guardian/next of kin for the publication of any potentially identifiable images or data included in this article.

## Author Contributions

GK contributed to the study design, data curation, and manuscript writing. GK, YY, and TM contributed to the interpretation of the results. GK, YY, KS, TY, TI, KO, and TM contributed to the investigation. All authors have reviewed and approved the final version of this manuscript.

## Funding

This study was supported by a grant from the MHLW Research program on rare and intractable diseases (grant number JPMH20FC1039).

## Conflict of Interest

The authors declare that the research was conducted in the absence of any commercial or financial relationships that could be construed as a potential conflict of interest.

## Publisher's Note

All claims expressed in this article are solely those of the authors and do not necessarily represent those of their affiliated organizations, or those of the publisher, the editors and the reviewers. Any product that may be evaluated in this article, or claim that may be made by its manufacturer, is not guaranteed or endorsed by the publisher.

## Abbreviations

ADC: apparent diffusion coefficient
AED: acute encephalopathy with reduced subcortical diffusion
AESD: acute encephalopathy with biphasic seizures and late reduced diffusion
AIEF: acute infantile encephalopathy predominantly affecting the frontal lobes
ALT: alanine aminotransferase
AST: aspartate aminotransferase
BUN: blood urea nitrogen
Crea: creatinine
DW: diffusion-weighted
fPHT: fosphenytoin
GCS: Glasgow Coma Scale
PCPC: Pediatric Performance Category Scale.

## References

[1] Takanashi J Oba H Barkovich AJ Tada H Tanabe Y Yamanouchi H. Diffusion MRI abnormalities after prolonged febrile seizures with encephalopathy. Neurology. (2006) 66:1304–9. 10.1212/01.wnl.0000210487.36667.a5 16682659

[2] Yadav SS Lawande MA Kulkarni SD Patkar DA. Acute encephalopathy with biphasic seizures and late reduced diffusion. J Pediatr Neurosci. (2013) 8:64–6. 10.4103/1817-1745.111429 23772250 PMC3680902

[3] Hayashi N Okumura A Kubota T Tsuji T Kidokoro H Fukasawa T. Prognostic factors in acute encephalopathy with reduced subcortical diffusion. Brain Dev. (2012) 34:632–9. 10.1016/j.braindev.2011.11.007 22177290

[4] Sakata K Kawano G Suda M Yokochi T Yae Y Imagi T. Determinants of outcomes for acute encephalopathy with reduced subcortical diffusion. Sci Rep. (2020) 10:1–11. 10.1038/s41598-020-66167-7 32499614 PMC7272444

[5] Yamanouchi H Kawaguchi N Mori M Imataka G Yamagata T Hashimoto T. Acute infantile encephalopathy predominantly affecting the frontal lobes. Pediatr Neurol. (2006) 34:93–100. 10.1016/j.pediatrneurol.2005.08.002 16458819

[6] Hoshino A Saitoh M Oka A Okumura A Kubota M Saito Y. Epidemiology of acute encephalopathy in Japan, with emphasis on the association of viruses and syndromes. Brain Dev. (2012) 34:337–43. 10.1016/j.braindev.2011.07.012 21924570

[7] Takanashi J. Two newly proposed infectious encephalitis/encephalopathy syndromes. Brain Dev. (2009) 31:521–8. 10.1016/j.braindev.2009.02.012 19339128

[8] Lee S Sanefuji M Torio M Kaku N Ichimiya Y Mizuguchi S. Involuntary movements and coma as the prognostic marker for acute encephalopathy with biphasic seizures and late reduced diffusion. J Neurol Sci. (2016) 370:39–43. 10.1016/j.jns.2016.09.018 27772782

[9] Niedermeyer E. Frontal lobe functions and dysfunctions. Clin Electroencephalogr. (1998) 29:79–90. 10.1177/155005949802900206 9571295

[10] Thompson PD. Frontal lobe ataxia. Handb Clin Neurol. (2012) 103:619–22. 10.1016/B978-0-444-51892-7.00044-9 21827922

[11] Terry JB Rosenberg RN. Frontal lobe ataxia. Surg Neurol. (1995) 44:583–8. 10.1016/0090-3019(95)00302-9 8669037

[12] Erasmus CE Beems T Rotteveel JJ. Frontal ataxia in childhood. Neuropediatrics. (2004) 35:368–70. 10.1055/s-2004-830370 15627946

[13] Tada H Takanashi JI Okuno H Kubota M Yamagata T Kawano G. Predictive score for early diagnosis of acute encephalopathy with biphasic seizures and late reduced diffusion (AESD). J Neurol Sci. (2015) 358:62–5. 10.1016/j.jns.2015.08.016 26333951

[14] Fiser DH. Assessing the outcome of pediatric intensive care. J Pediatr. (1992) 121:68–74. 10.1016/S0022-3476(05)82544-2 1625096

[15] Ogutu BR Newton CR Muchohi SN Otieno GO Edwards G Watkins WM. Pharmacokinetics and clinical effects of phenytoin and fosphenytoin in children with severe malaria and status epilepticus. Br J Clin Pharmacol. (2003) 56:112–9. 10.1046/j.1365-2125.2003.01829.x 12848783 PMC1884335

[16] Robinson J Morris B Aherne G Marks V. Pharmacokinetics of a single dose of phenytoin in man measured by radioimmunoassay. Br J Clin Pharmacol. (1975) 2:345–9. 10.1111/j.1365-2125.1975.tb02782.x 1233994 PMC1402591

[17] Aweeka FT Gottwald MD Gambertoglio JG Wright TL Boyer TD Pollock AS. Pharmacokinetics of fosphenytoin in patients with hepatic or renal disease. Epilepsia. (1999) 40:777–82. 10.1111/j.1528-1157.1999.tb00778.x 10368078

[18] Pfizer. CEREBYX^®^, Dosage And Administration. Available online at: https://www.pfizermedicalinformation.com/en-us/cerebyx/dosage-admin (accessed 9 October, 2020).

[19] Fischer JH Patel TV Fischer PA. Fosphenytoin: clinical pharmacokinetics and comparative advantages in the acute treatment of seizures. Clin Pharmacokinet. (2003) 42:33–58. 10.2165/00003088-200342010-00002 12489978

[20] Shaikh AS Li Y Cao L Guo R. Analysis of phenytoin drug concentration for evaluation of clinical response, uncontrolled seizures and toxicity. Pak J Pharm Sci. (2018) 31:1697–700. 30203765

[21] International Programme on Chemical Safety. Poisons Information Monograph 416. (1997). Available online at: http://www.inchem.org/documents/pims/pharm/pim416.htm/ (accessed 10 October, 2020).

[22] Poretti A Christen HJ Elton LE Baumgartner M Korenke GC Sukhudyan B. Horizontal head titubation in infants with Joubert syndrome: a new finding. Dev Med Child Neurol. (2014) 56:1016–20. 10.1111/dmcn.12489 24814865

[23] Tully HM Dempsey JC Ishak GE Adam MP Mink JW Dobyns WB. Persistent figure-eight and side-to-side head shaking is a marker for rhombencephalosynapsis. Mov Disord. (2013) 28:2019–23. 10.1002/mds.25634 24105968 PMC5510988

[24] Takanashi J Sugita K Barkovich AJ Takano H Kohno Y. Partial midline fusion of the cerebellar hemispheres with vertical folia: a new cerebellar malformation? AJNR Am J Neuroradiol. (1999) 20:1151–3. 10445461 PMC7056222

[25] Bruns L. Ueber storungen des gleichgewichtes bei stirnhirntumoren. Dtsch Med Wchnschr Leipz. (1892) 18:138–40. 10.1055/s-0029-1198943

[26] Sala DS Francescani A Spinnler H. Gait apraxia after bilateral supplementary motor area lesion. J Neurol Neurosurg Psychiatry. (2002) 72:77–85. 10.1136/jnnp.72.1.77 11784830 PMC1737704

[27] Gordon N. Ataxia of parietal lobe origin. Dev Med Child Neurol. (1999) 41:353–5. 10.1017/S0012162299000778 10378764

[28] Kown YC Kim JH Ahn TB. Ataxia of cortical origin via crossed cerebellar diaschisis. Neurol Sci. (2015) 36:161–3. 10.1007/s10072-014-1846-x 24899224

[29] Scoppa F Capra R Gallamini M Shiffer R. Clinical stabilometry standardization: basic definitions - acquisition interval-sampling frequency. Gait Posture. (2013) 37:290–2. 10.1016/j.gaitpost.2012.07.009 22889928

[30] Almutairi A Christy JB Vogtle L. Vestibular and oculomotor function in children with cerebral palsy: a scoping review. Semin Hear. (2018) 39:288–304. 10.1055/s-0038-1666819 30038456 PMC6054580

[31] Okumura A Kidokoro T Tsuji T Suzuki M Kubota T Kato T. Differences of clinical manifestations according to the patterns of brain lesions in acute encephalopathy with reduced diffusion in the bilateral hemispheres. Am J Neuroradiol. (2009) 30:825–30. 10.3174/ajnr.A1431 19131408 PMC7051762

[32] Yokochi T Takeuchi T Mukai J Akita Y Nagai K Obu K. Prediction of acute encephalopathy with biphasic seizures and late reduced diffusion in patients with febrile status epilepticus. Brain Dev. (2016) 38:217–24. 10.1016/j.braindev.2015.07.007 26242200

