# Supplementary material for: Truncal Instability and Titubation in Patients With Acute Encephalopathy With Reduced Subcortical Diffusion
Source: Front Neurol. 2021 Sep 17;12:740655. doi: 10.3389/fneur.2021.740655 (PMC8484920; doi:10.3389/fneur.2021.740655)
Supplement: Supplementary file 3 [file Presentation_1.PPTX]

## Slide 1
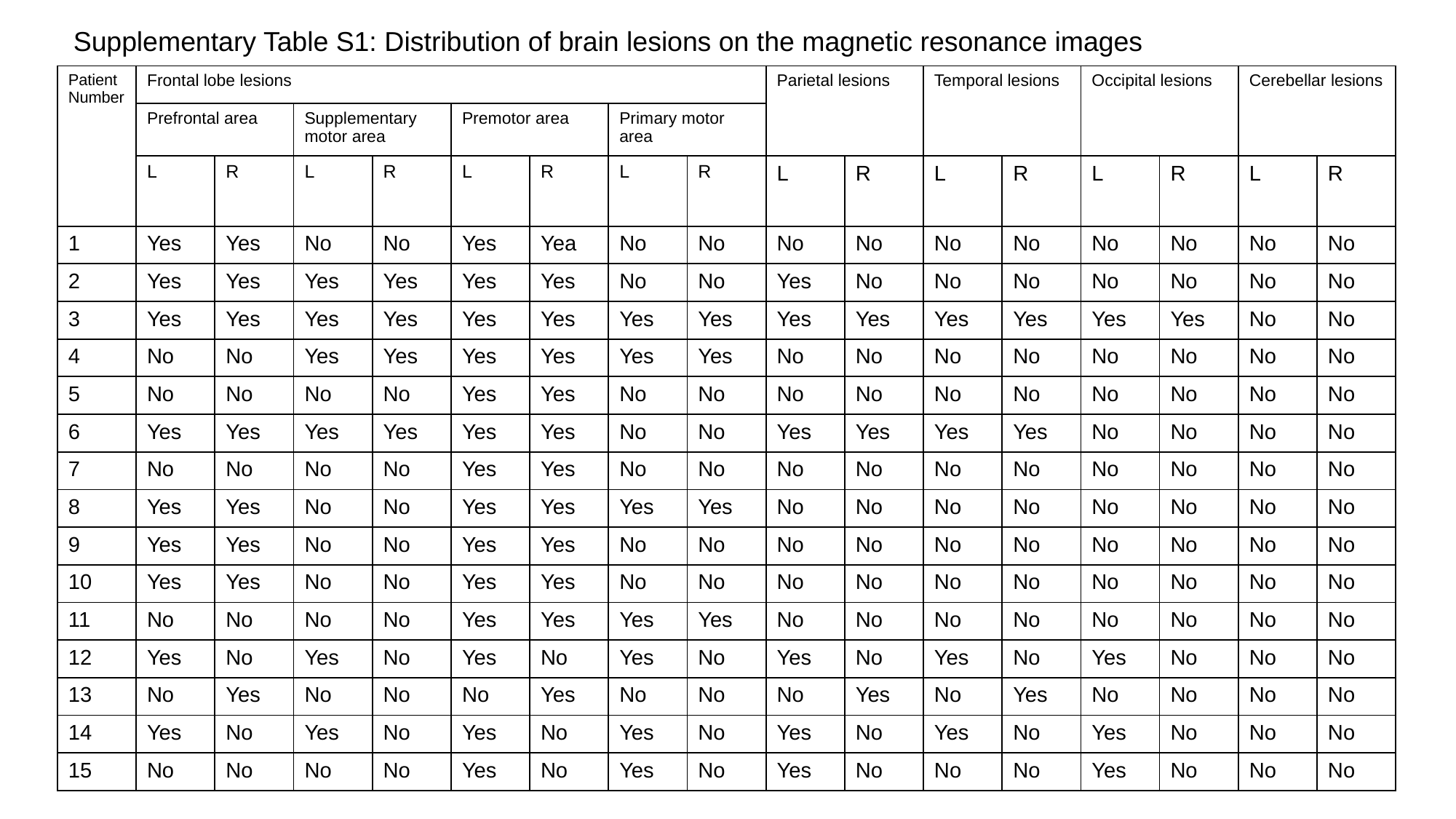

Supplementary Table S1: Distribution of brain lesions on the magnetic resonance images
| Patient Number | Frontal lobe lesions | | | | | | | | Parietal lesions | | Temporal lesions | | Occipital lesions | | Cerebellar lesions | |
| --- | --- | --- | --- | --- | --- | --- | --- | --- | --- | --- | --- | --- | --- | --- | --- | --- |
| | Prefrontal area | | Supplementary motor area | | Premotor area | | Primary motor area | | | | | | | | | |
| | L | R | L | R | L | R | L | R | L | R | L | R | L | R | L | R |
| 1 | Yes | Yes | No | No | Yes | Yea | No | No | No | No | No | No | No | No | No | No |
| 2 | Yes | Yes | Yes | Yes | Yes | Yes | No | No | Yes | No | No | No | No | No | No | No |
| 3 | Yes | Yes | Yes | Yes | Yes | Yes | Yes | Yes | Yes | Yes | Yes | Yes | Yes | Yes | No | No |
| 4 | No | No | Yes | Yes | Yes | Yes | Yes | Yes | No | No | No | No | No | No | No | No |
| 5 | No | No | No | No | Yes | Yes | No | No | No | No | No | No | No | No | No | No |
| 6 | Yes | Yes | Yes | Yes | Yes | Yes | No | No | Yes | Yes | Yes | Yes | No | No | No | No |
| 7 | No | No | No | No | Yes | Yes | No | No | No | No | No | No | No | No | No | No |
| 8 | Yes | Yes | No | No | Yes | Yes | Yes | Yes | No | No | No | No | No | No | No | No |
| 9 | Yes | Yes | No | No | Yes | Yes | No | No | No | No | No | No | No | No | No | No |
| 10 | Yes | Yes | No | No | Yes | Yes | No | No | No | No | No | No | No | No | No | No |
| 11 | No | No | No | No | Yes | Yes | Yes | Yes | No | No | No | No | No | No | No | No |
| 12 | Yes | No | Yes | No | Yes | No | Yes | No | Yes | No | Yes | No | Yes | No | No | No |
| 13 | No | Yes | No | No | No | Yes | No | No | No | Yes | No | Yes | No | No | No | No |
| 14 | Yes | No | Yes | No | Yes | No | Yes | No | Yes | No | Yes | No | Yes | No | No | No |
| 15 | No | No | No | No | Yes | No | Yes | No | Yes | No | No | No | Yes | No | No | No |

## Slide 2
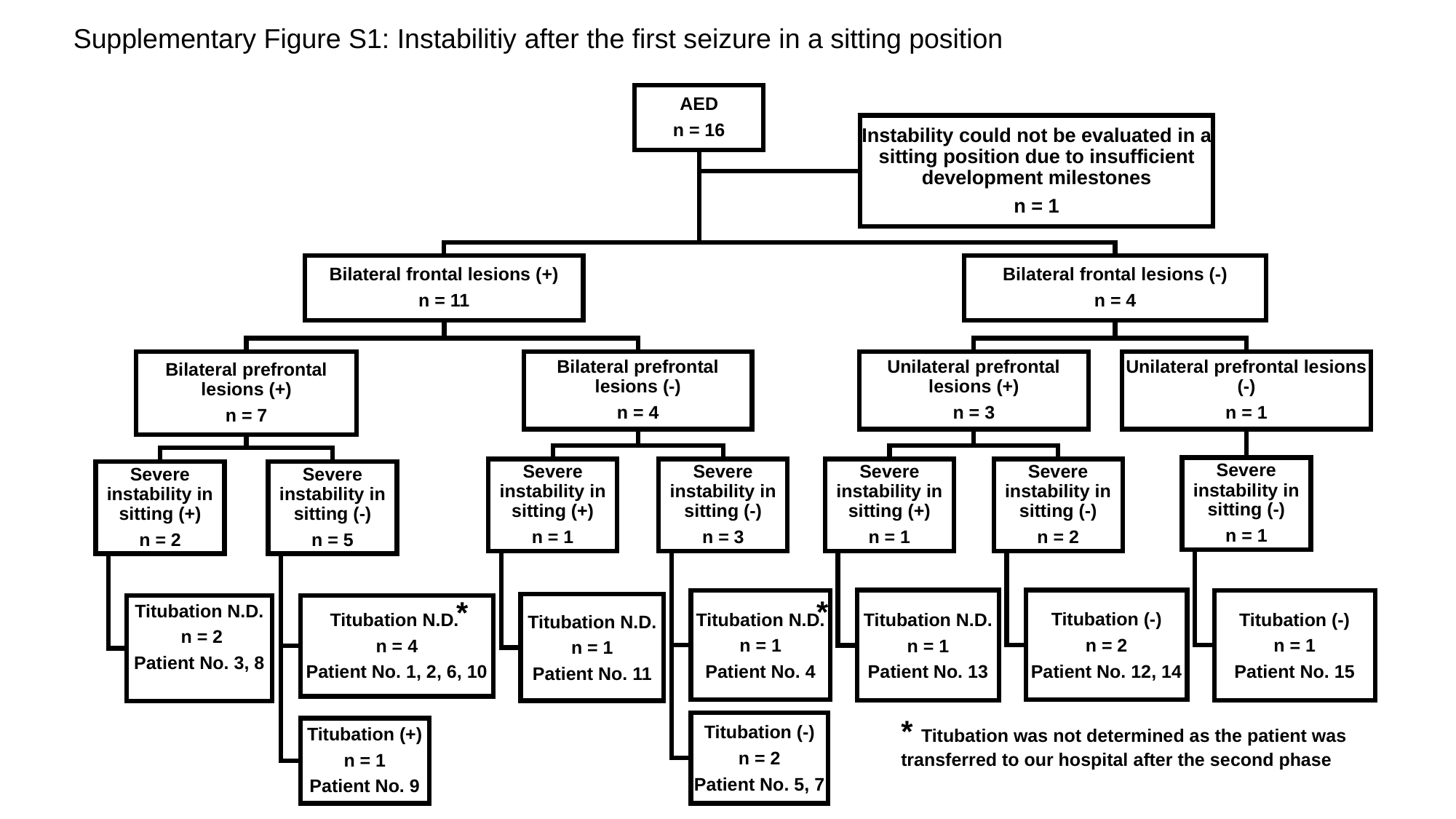

Supplementary Figure S1: Instabilitiy after the first seizure in a sitting position
*
*
* Titubation was not determined as the patient was transferred to our hospital after the second phase

## Slide 3
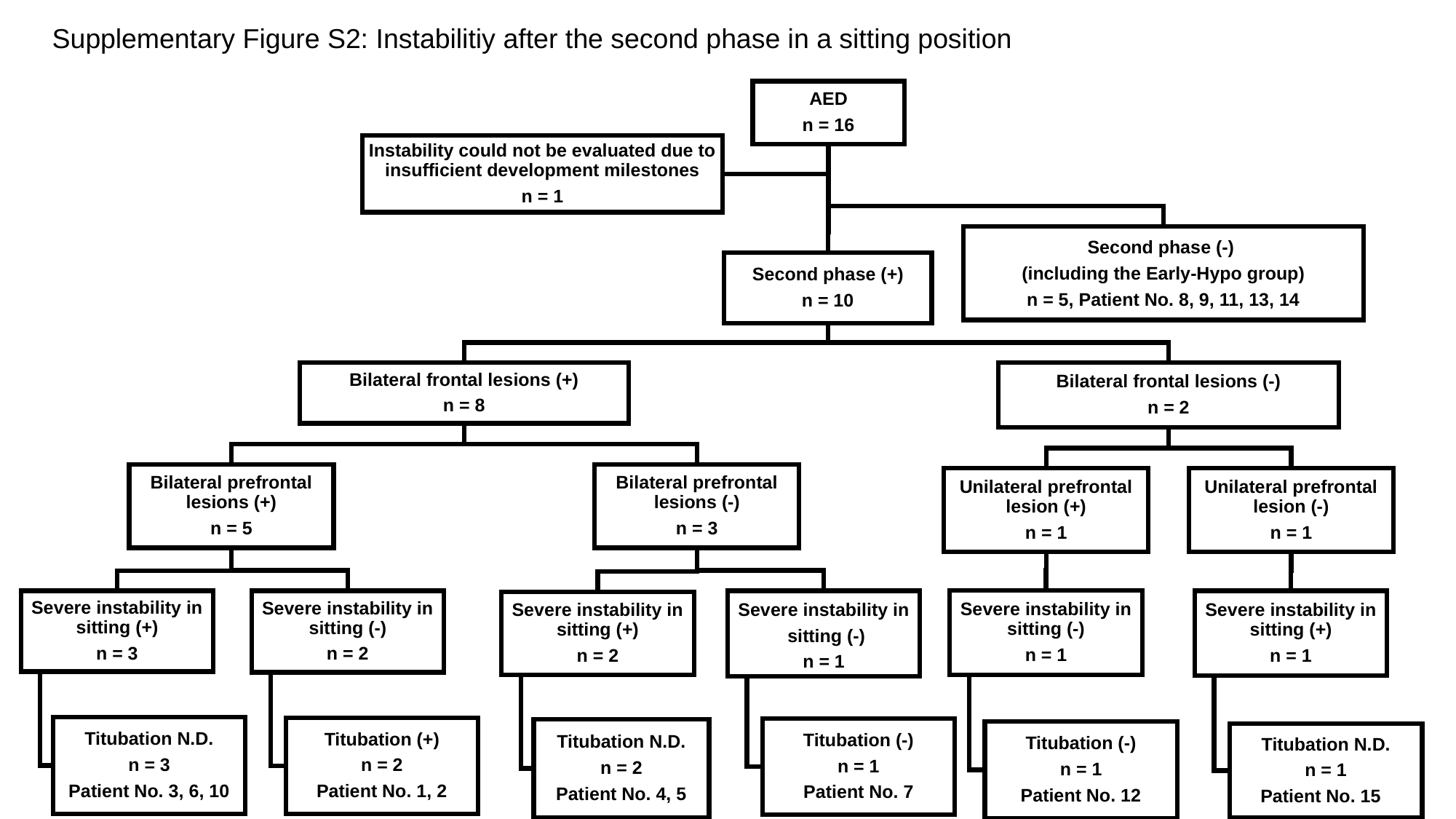

Supplementary Figure S2: Instabilitiy after the second phase in a sitting position

## Slide 4
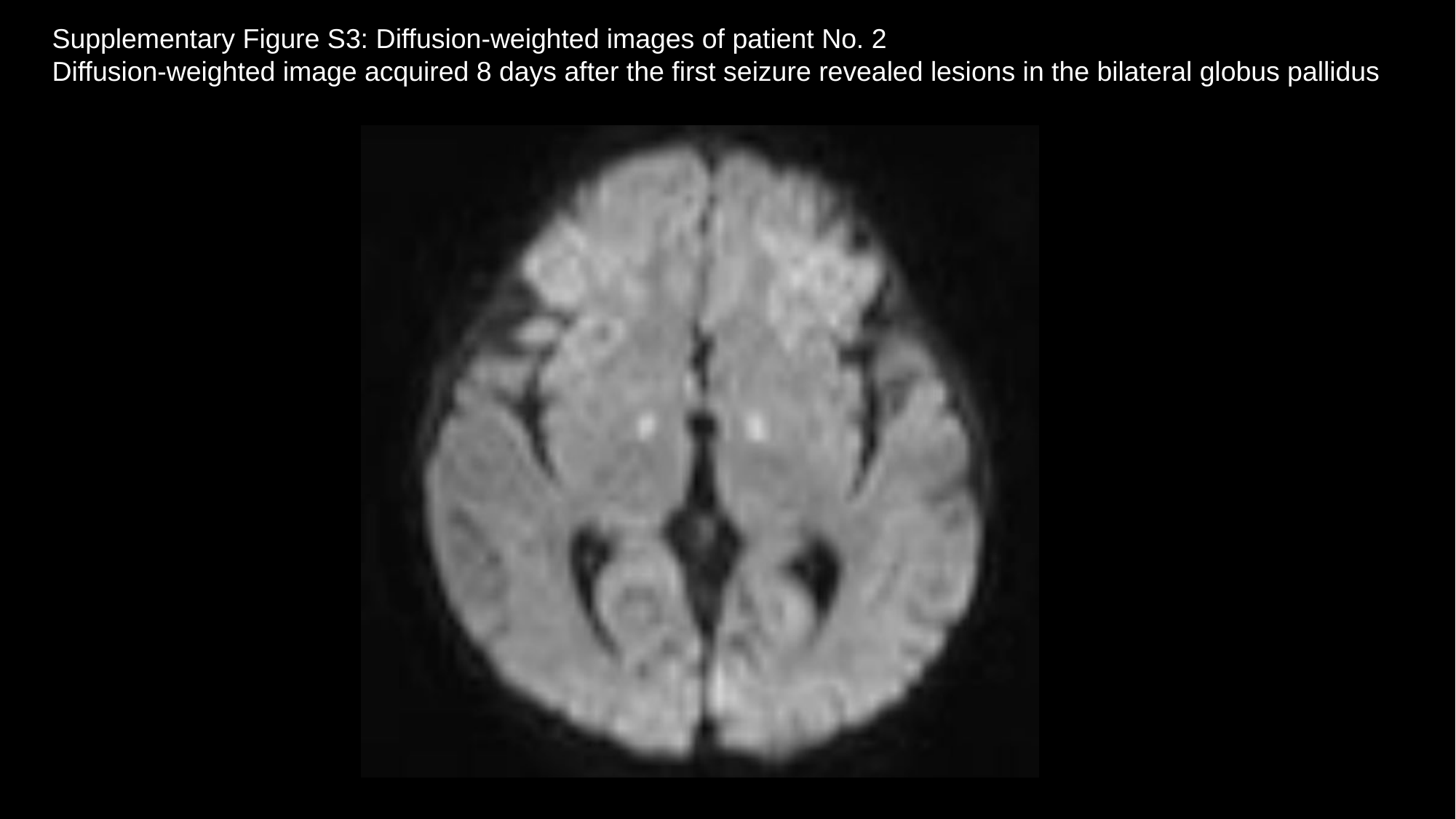

Supplementary Figure S3: Diffusion-weighted images of patient No. 2
Diffusion-weighted image acquired 8 days after the first seizure revealed lesions in the bilateral globus pallidus
